# Acceptance of Supportive Illustrations for Preparation of Patients for an Orthopedic Telemedical Consultation

**DOI:** 10.3389/fsurg.2021.696721

**Published:** 2021-09-22

**Authors:** Katharina Estel, Gordian Weber, Luise Richter, Marko Hofmann, Patrick Ruckdeschel, Sven Märdian, Christian Willy, David Alexander Back

**Affiliations:** ^1^Clinic for Traumatology and Orthopedics, Bundeswehr Hospital Berlin, Berlin, Germany; ^2^Medical Faculty of the Charité - Universitätsmedizin Berlin, Berlin, Germany; ^3^Institute for Computer Engineering Faculty of Computer Science, University of the Bundeswehr Munich, Neubiberg, Germany; ^4^Center for Musculoskeletal Surgery, Charité - Universitätsmedizin Berlin, Berlin, Germany; ^5^Dieter Scheffner Center for Medical Education and Educational Research, Charité – Universitätsmedizin Berlin, Berlin, Germany

**Keywords:** illustration, telemedicine, preparation, orthopedics, patients

## Abstract

**Introduction:** Telemedical video consultations are a powerful support for patient–doctor interactions. For optimal digital settings, explanatory illustrations may be helpful for patients. This study analyzed patients' the attitude of patients to illustrations preparing for an orthopedic telemedical consultation (OTC).

**Methods:** A leaflet with eight illustrations was designed and their acceptance and estimated necessity was evaluated among patients who had experienced an OTC (EXP-group) and others who had not (NOV-group) with a 12-item-questionnaire.

**Results:** Sixty patients participated (*n* = 30 each group). All illustrations were evaluated positively. The EXP-group gave significantly higher ratings than the NOV-group for improved understanding by the given keywords of the illustrations (*p* = 0.046), preference for being informed by illustrations than by merely by a pure text (*p* = 0.023), better feeling of preparation for an OTC by the illustrations (*p* = 0.005), and the impression of a simplified process of the OTC by the illustrations (*p* = 0.012).

**Discussion:** While the illustrations were well-accepted by the participants, significant differences were revealed between the valuation of single aspects by patients, depending on a previous experience with an OTC. Therefore, a leaflet with explanatory illustrations may be helpful in preparing patients for an OTC to support the digital patient–doctor contact.

## Introduction

Digital transformation of the healthcare sector has accelerated many promising innovations (1). Among these, telemedicine can help to provide professional patient care in structurally weak regions, if internet capacities are sufficient (2). The use of digital tools may also reduce costs and time expenditure for both patients and staff (3–5). Telemedicine can be verifiably supportive in the primary care of patients (6), in surgery for the assessment of wounds (7), or postoperative follow-up (8). Still, technical problems, a lack of computer skills, or apathy of the users may pose barriers to its success (9). However, the current Sars-COV-19 pandemic forced the development of new concepts of contactless treatment options, increasing the importance and acceptance of telemedical solutions. Direct patient contacts in medical practices and hospitals could be reduced and infection chains minimized. With the use of telemedicine, new helpful strategies can be implemented, and thus patients and staff can be protected (10).

Telemedical consultations will be carried out differently, depending on the medical discipline. Specialties, such as psychiatry, work successfully with a conversational psychotherapy (11). Others, such as orthopedics, depend largely on physical examinations or radiological findings for defining individual treatment strategies (2). However, recent studies have already shown that functional examinations of the hip, knee, shoulder or elbow joints, for example, can also be carried out effectively by means of telemedicine (12–14).

For the successful performance of a telemedical consultation, participants must fulfill relevant prerequisites, both hard-skills (handling appropriate technology) and soft-skills (knowledge of the process) (9). Thus, it might be helpful to introduce essential information to the patients in advance to gain acceptance for this digital form of interaction. Acceptance of a telemedical consultation compared to a live consultation by patients is described very differently in literature. Some studies reported a very positive attitude of patients toward telemedical consultations in trauma surgery due to lower costs, reduced travel time, or less need for childcare (15), whereas other groups found contrary results (16). However, a positive attitude toward telemedicine is an essential prerequisite for good digital communication between physicians and patients, and it can be increased essentially by knowledge about the processes (9, 17). Illustrations explaining the procedure have already been established in telemedicine to increase the understanding and acceptance of patients (18). Furthermore, it could be shown that complicated medication plans are better understood with the help of illustrations (19, 20). In patients with infectious or chronic diseases, illustrations were able to support the adherence to instructions on specific behaviors (20, 21). Illustrations have also proven to be helpful in preparation before an intervention, also concerning the incidence and severity of consequences or complications of the intervention (17). However, data regarding the usefulness of illustrations concerning telemedical consultations in orthopedic and trauma surgery are still lacking.

This study aimed to analyze the acceptance of explanatory illustrations among patients in preparation for an orthopedic telemedical consultation (OTC). A particular point of interest was, whether there would be a difference in the appraisal of single illustrations or information depending on the fact, if patients had already undergone a telemedical consultation or not.

## Materials and Methods

### Study Design

This study was conducted from April 2020 through June 2020 in the Clinic for Trauma Surgery and Orthopedics of the Bundeswehr Hospital, Berlin. The study cohort was divided into two groups depending on whether or not they had at least one OTC experience in the past (group 1: Experienced = EXP, group 2: Novice = NOV). To evaluate the study hypotheses, patients were asked to complete a 12-item-questionnaire delivered to all participants as hard copies (Supplementary 1). The questionnaire was designed as described below. When invited to the study, participants were informed in advance about the content of the survey, granted anonymity, and voluntary participation. The responsible ethics committee approved the study (No. Eth-12/19).

### Development of the Illustrations

**Figure 1:**
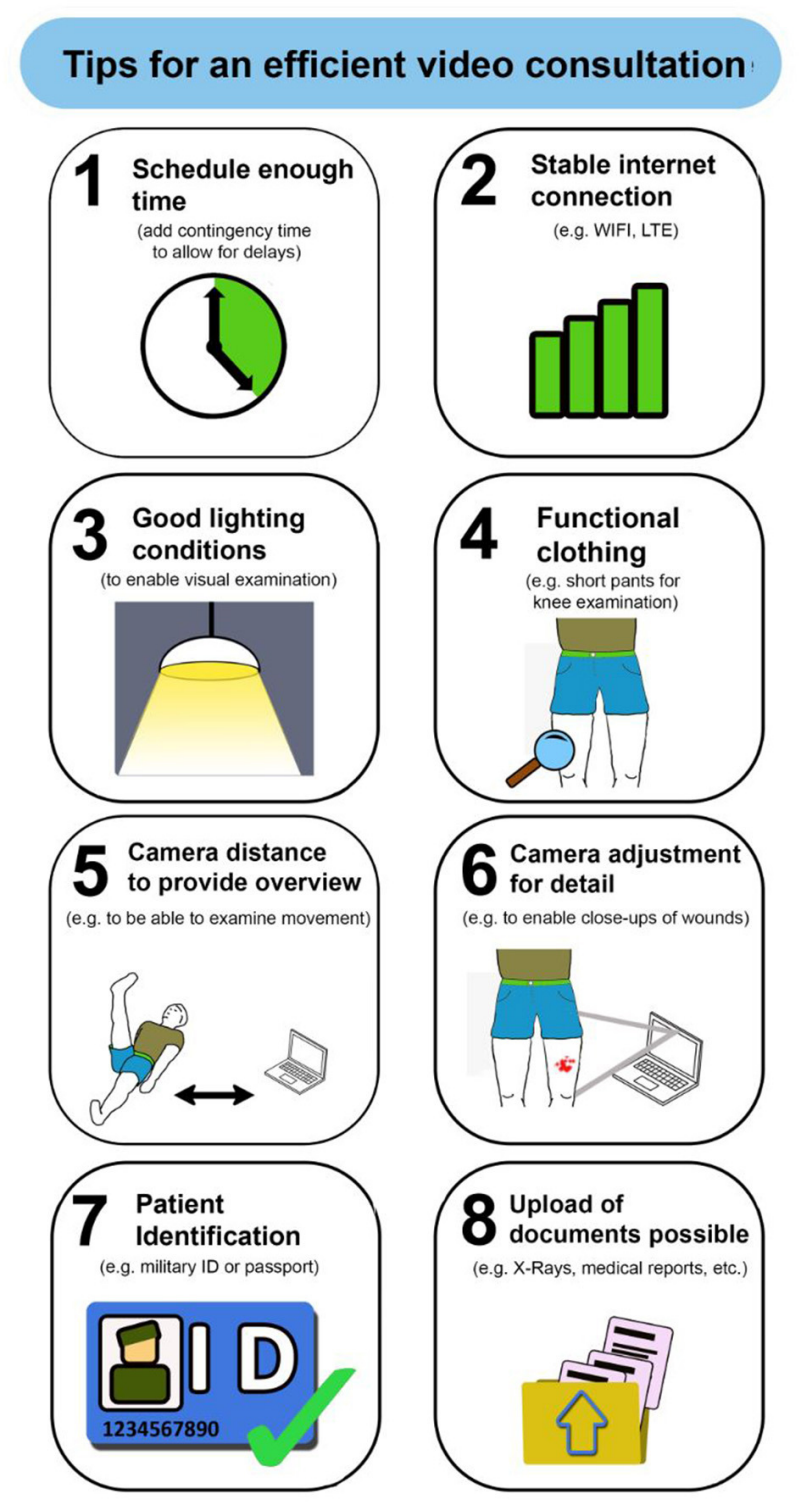
Leaflet with the illustrations as preparation for an orthopedic telemedical consultation.

In the run-up to the project, illustrations with numbering from 1 to 8 were designed in cooperation with the Institute for Computer Engineering at the Faculty of Computer Science at the Bundeswehr University in Munich, Germany. To provide patients with important information about an OTC, illustrations and keywords were used to facilitate the preparation for and measures during an OTC (see Figure 1).

### Design of the Questionnaire

Based on the illustrations, 12 questions were defined. The questions were divided into four groups that addressed the following areas of interest:

1. Sociodemographic data [gender, age; (two questions with one open question and one yes/no-answer].
2. General questions about the illustrations (three questions), about the usefulness of the illustrations (yes/no-answers, possible open answers), the relevance of each illustration (categorized from “1 = very good” to “6 = insufficient,” according to the German school system), and aspects that participants had not considered before an OTC (Exp-group only, yes/no-answers, possible open answers).
3. Six specific questions (five Likert-scaled questions from “1 = fully agree” to “5 = fully disagree”) on comprehensibility of illustrations, supporting effect of the keywords used, comprehensibility of the illustrations compared to pure text form, potential help in preparation for an OTC, simplification of the process of the OTC, and appealing design.
4. Finally, a concluding open question asked about possible improvements of the leaflet with its explanatory illustrations (one question).

### Data Analysis

A quantitative analysis of the data was performed using descriptive statistical means using Microsoft Excel® (Microsoft Corp., Redmond, WA, USA) (Supplementary 2). For the age, mean value (MV) and standard deviation (±) were determined. For statistical analyses, SPSS (version 2020, IBM Corp., Armonk, NY, USA) was used. Student's *t*-test was applied for comparison of the answers in the scaled questions. The level of significance was set to *p* < 0.05. Two independent reviewers screened free-text answers for repetitive sequences.

## Results

### Sociodemographic Data

A total of 60 patients participated in the survey (EXP: *n* = 30, NOV: *n* = 30), 54 were male (EXP: *n* = 28, NOV: *n* = 26) and six female (EXP: *n* = 2, NOV: *n* = 4). The participants were on the average 38.7 (± 11.0) years of age (EXP: 38.5 ± 9.5; NOV: 38.9; ± 12.3, *p* = 0.899).

### General Questions About the Illustrations

Asked for the perceived usefulness of the illustrations, 48 patients answered with “yes” (EXP: *n* = 22, NOV: *n* = 26; *p* = 0.203). In this respect, the following figures were, in particular, rated highest: “functional clothing” (*n* = 17; EXP: *n* = 3, NOV: *n* = 14), “camera adjustment for detail” (*n* = 16; EXP: *n* = 5, NOV: *n* = 11), “camera distance to provide overview” (*n* = 16; EXP: *n* = 3, NOV: *n* = 13), and “good light conditions” (*n* = 14; EXP: *n* = 3, NOV: *n* = 11) (multiple answers possible). Additionally, 15 patients of the whole group (EXP: *n* = 7, NOV: *n* = 8) mentioned particularly that all illustrations could be useful with regard to an upcoming telemedical consultation.

**Figure 2:**
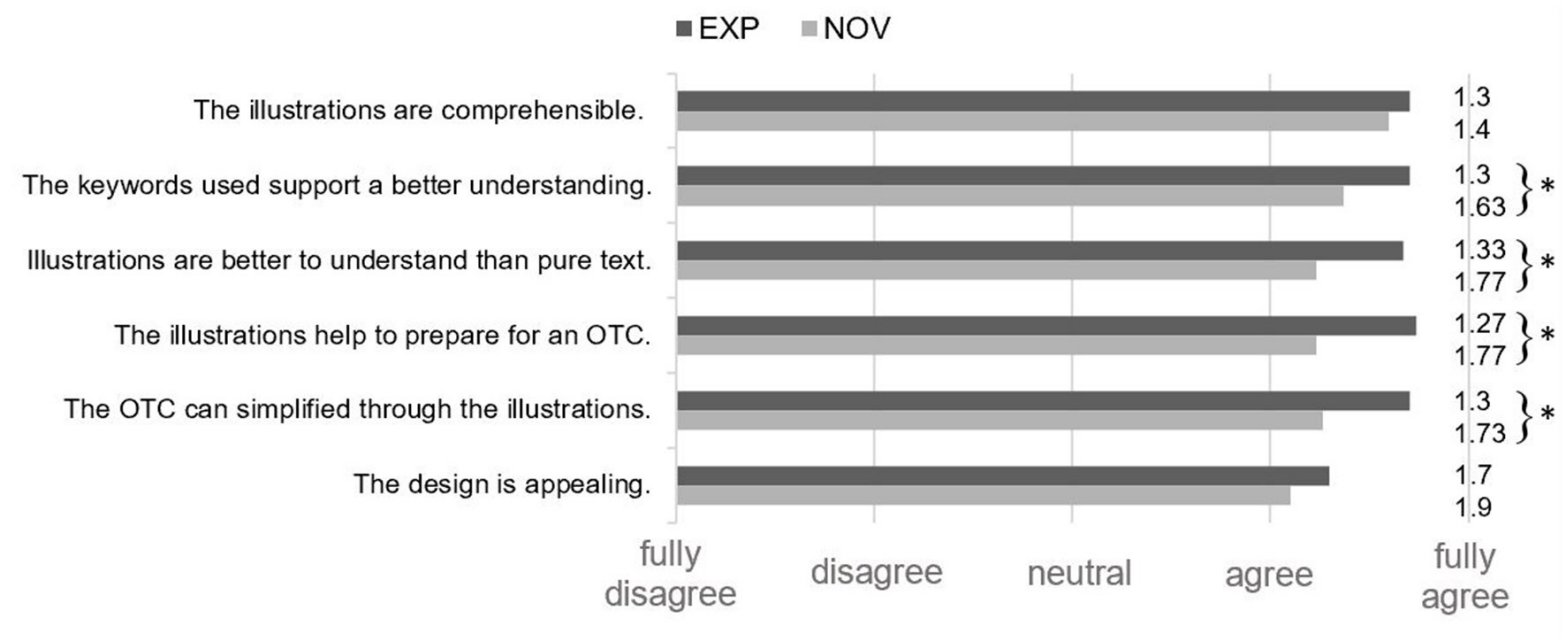
Presentation of the results of assessment of the characteristics by the patients of the illustrations using the Likert Scale (1 - fully agree to 5 - fully disagree) (EXP *n* = 30, NOW *n* = 30) (*p* < 0.05). *Significant differences were demonstrated for these results.

Figure 2 shows the perceived relevance of each illustration. The content “Stable internet connection” was considered most important by both groups (mean value EXP: 1.03; NOV: 1.07). The necessity of a “camera adjustment for detail” still received high ratings from both groups, but least compared to the other illustrations (mean value EXP: 2.00; NOV: 2.33). There were no significant differences between both groups.

Ten participants of the EXP-group agreed that there had been aspects depicted on the illustration that they had not considered before using an OTC. Especially the illustrations with the content “camera distance to provide overview” (EXP: *n* = 6), “camera adjustment for detail” (EXP: *n* = 6), and “good light conditions” (EXP: *n* = 5) were named (multiple answers possible).

### Specific Questions About the Illustrations

**Figure 3:**
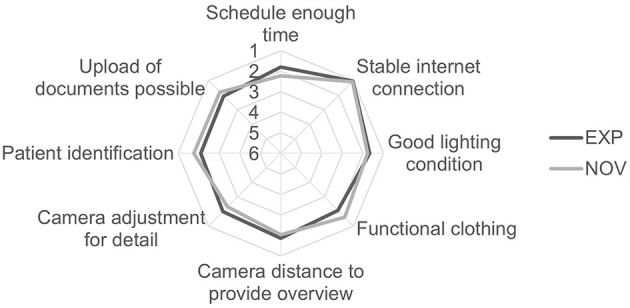
Results of the assessment of the perceived relevance of each individual illustration (rating according to the German School grading system from “1 – very good” to “6 – Insufficient”) (EXP *n* = 30, NOV *n* = 30).

Figure 3 shows the assessment of characteristics by the patients of the illustrations using the Likert Scale. The highest agreement could be achieved concerning the comprehensibility of the illustrations (MV: EXP: 1.3; NOV: 1.4; *p* = 0.484). The design evaluation received comparatively lowest score, but was still highly ranked by both the groups (MV: EXP: 1.7; NOV: 1.9; *p* = 0.339). However, the EXP-group ranked different aspects significantly higher than the NOV-group, particularly the supporting effect of the keywords used (*p* = 0.012), the comparably better understanding than pure text form (*p* = 0.005), the potential help to prepare for an OTC (*p* = 0.023), and the simplification of the process of the OTC through the illustrations (*p* = 0.046).

In the concluding open question eight patients of the EXP-group and seven patients of the NOV-group gave helpful comments. The proposed improvements of the group encompassed the adjustment or omitting individual illustrations (*n* = 9), the optimization of the pictorial representation (*n* = 5), and the optimization of the key words used (*n* = 1).

## Discussion

In the context of the current digital transformation of the healthcare system, telemedicine is becoming increasingly important. Taking into account some important circumstances in advance like existing technology and computer skills, an ‘OTC can be a satisfactory experience for both patients and doctors (9). While physicians will quickly gain experience with their OTCs, it might be helpful to prepare patients for OTCs to ensure a positive attitude and finally also a good digital interaction (18). In this background, the presented study investigated the acceptance of explanatory illustrations among orthopedic patients as preparation for an OTC. To assess the perception of the given illustrations from different points of view, two groups of participants were included. The first group had experienced an OTC before and the second group had no prior experience with an OTC.

In the survey, most respondents found the illustrations **useful** and **helpful as preparation** for an OTC. This positive aspect of explanatory illustrations as preparation for a video consultation could have been expected as a similarly high level of acceptance of informative illustrations could be demonstrated among patients in several studies (22–25). But there are also studies with different results regarding the acceptance of the provision of information prior to a medical consultation (15, 16). Also, practical medical impacts could be shown, when for example, preoperative illustrations before a catheterization of the urinary bladder could reduce the incidence and the severity of catheter-related bladder discomfort (17). As even the NOV-group stated that they could imagine the illustrations to be helpful for the preparation for an assumed OTC, this may support a potential success of such offers on patients, regardless of a prior OTC experience (15).

In the presented study, the EXP-group rated the supporting effect of the **keywords** used and the potential help to prepare for an OTC significantly higher than the NOV-group. This may be explained by the fact that patients who had experienced an OTC knew what information contained in the keywords would have helped them in relation to the OTC.

With regard to the preferred form of the presented information, it has already been shown that conveying information through cartoons is a more effective method for providing medical information than through a pure text form (26). A similar tendency could be observed in the presented study, in which the EXP-group evaluated the potential understanding by illustrations as by pure text form significantly higher than the reference group.

Moreover, the EXP-group indicated that the illustrations could simplify the OTC. This will be most likely based on the knowledge conveyed by the illustrations. Increased knowledge can in turn have a positive effect on the doctor–patient communication with less uncertainties regarding an OTC (9, 17). Since the two groups differed significantly in this respect, it may be postulated that the Nov-group believed that an OTC was simple to conduct even without prior information. But the own results showed that even one third of the Exp-group did not sufficiently consider one or more of the **aspects depicted on the illustrations** before using an OTC.

The **differences between the two groups suggest that** that an OTC with previous experience with this medium may be assessed differently than one without that experience. In particular, the preparation and process may be underestimated by OTC inexperienced patients. The positive evaluations of the EXP-group showed, however, that leaflets can certainly find their justification in the preparation of OTC. We assume that a pronounced knowledge of the process will make the doctor–patient communication and thus the OTC more successful.

It has already been shown that a low level of education, too high expectations of the user toward the telemedicine, or apathy pose relevant patient-related obstacles for the adoption of telemedical consultations (9). If these obstacles could be diminished by the previous use of a leaflet, telemedical counseling could be further improved as a relevant supportive instrument of digitalized medicine.

The study population gave some **helpful comments to improve** their own leaflet, which will be used for its further development. **For other medical fields** a leaflet can of course vary greatly in terms of design and the number of illustrations. Depending on the question, there are many possibilities to inform patients.

Among the **limitations** of the study is the small number of participants and the fact, that the patients are quite young on the average, which might impede representative conclusions to be drawn from the chosen patient population to elderly patients. Furthermore, there was no randomization of the participants. The voluntary nature of participation could also have resulted in a biased selection, with participants who either experienced an OTC or were interested in digital topics such as telemedicine might have reacted positively to the images.

In future, even more patients of different age groups should be evaluated to exclude a possible internet affinity bias of the younger generation. The appreciation and attitude of the physician should also be valued. Moreover, data presented here should be checked in multicenter studies with larger samples and also in direct connection to measurable improvements of the OTC performances. On the basis of these data, it can be determined whether a leaflet would be useful to prepare patients for a telemedical consultation.

## Conclusion

In summary, based on the results of the presented study with some significant differences between the two included patient groups, it may be advisable to distribute a leaflet regularly before an OTC in order to better prepare and improve the process itself. In addition, the results of the two groups also showed that certain aspects may only be seen with the experience of a completed OTC, and should therefore be precisely explained in a medical consultation with regard to underline their importance. The leaflet should be continuously developed based on the comments of the respondents, and used in practical and also multicenter digital medical treatment approaches.

## Data Availability Statement

The raw data supporting the conclusions of this article will be made available by the authors, without undue reservation.

## Ethics Statement

The studies involving human participants were reviewed and approved by the responsible ethics committee - Ärztekammer Berlin - approved the study (No. Eth-12/19). The patients/participants provided their written informed consent to participate in this study.

## Author Contributions

KE and LR participation in the development of the concept, design and implementation of the study, analysis of the data, and preparation of the paper. GW, CW, and DB participation in the development of the concept, design and implementation of the study, analysis of the data, and preparation of the paper. MH and PR participation in the development and preparation of the flier and participation in the preparation of the paper. All authors contributed to the article and approved the submitted version.

## Funding

The funding of the entire project (No. 23K4-S-10 1921) orthopedic telemedical consultation was granted by the Medical Service of the German Armed Forces.

## Conflict of Interest

The authors declare that the research was conducted in the absence of any commercial or financial relationships that could be construed as a potential conflict of interest.

## Publisher's Note

All claims expressed in this article are solely those of the authors and do not necessarily represent those of their affiliated organizations, or those of the publisher, the editors and the reviewers. Any product that may be evaluated in this article, or claim that may be made by its manufacturer, is not guaranteed or endorsed by the publisher.
