# Supplementary material for: Acceptance of Supportive Illustrations for Preparation of Patients for an Orthopedic Telemedical Consultation
Source: Front Surg. 2021 Sep 22;8:696721. doi: 10.3389/fsurg.2021.696721 (PMC8492955; doi:10.3389/fsurg.2021.696721)
Supplement: Supplementary file 1 [file Table_1.DOCX]

| **To the**  **[Journal Frontiers in Surgery](https://home.liebertpub.com/publications/telemedicine-and-e-health/54)**  **Editorial Board**  **PD Dr. med. Koroush Kabir** |  |
| --- | --- |

Berlin, 16.04.2021

Dear PD Dr. med. Koroush Kabir,

,

We would like to submit the attached manuscript as an original research article to your journal entitled “**Acceptance of supportive illustrations for patients’ preparation for an orthopedic telemedical consultation**”.

In our study, we investigated the acceptance of explanatory illustrations among orthopedic patients as preparation for an OTC. We believe that an orthopedic telemedical consultation (OTC) can be a satisfactory experience for both patients and doctors. Therefore, it might be helpful to prepare patients for OTCs with explanatory illustration to ensure a positive attitude and finally also a good digital interaction.

After a flyer with 8 explanatory illustrations was created, 30 participants with experience of an OTC and 30 participants without experience of an OTC were asked about acceptance and benefits.

Results indicated that all illustrations were positively evaluated. Certain illustrations were also rated significantly better by participants with OTC experience. This shows that certain aspects are only seen with the experience of a completed OTC and therefore these aspects should be explained in detail in the medical discussion with regard to underline their importance.

Given the enormous importance of online video consultations, especially in this era of the Sars-Cov-2 pandemic, we believe that our paper with demonstrated positive impact of explanatory illustrations to help patients prepare for an OTC falls within the scope of your journal and would be very pleased if you would consider it for publication.

Yours sincerely,

K.E.
