# Supplementary material for: Acceptance of Supportive Illustrations for Preparation of Patients for an Orthopedic Telemedical Consultation
Source: Front Surg. 2021 Sep 22;8:696721. doi: 10.3389/fsurg.2021.696721 (PMC8492955; doi:10.3389/fsurg.2021.696721)
Supplement: Supplementary file 2 [file Table_2.DOCX]

| **1.** | **Sociodemographic data** |
| --- | --- |
|  | 1. Age? ___ years 2. Gender : ⬜ female ⬜ male |

| **2.** | **General questions about the illustrations** |
| --- | --- |
|  | 1. Looking back, would you find it useful if you had received the flyer with the illustrations before your online video consultation?   ⬜ Yes ⬜ No  If yes, on which points? _______________________________________________________________________________________   1. How would you rate the individual aspects depicted on the flyer from your own experience with school grades from 1-6 (Reference to the German school system)?   **("1 - very useful/important" to "6 - not at all useful/important")**  1 – Schedule enough time 1 2 3 4 5 6  2 – Stable internet connection 1 2 3 4 5 6  3 – Good lighting condition 1 2 3 4 5 6  4 – Functional clothing 1 2 3 4 5 6  5 – Camera distance to provide overview 1 2 3 4 5 6  6 – Camera adjustment for detail 1 2 3 4 5 6  7 – Patient identification 1 2 3 4 5 6  8 – Upload of documents possible 1 2 3 4 5 6   1. Did you not sufficiently consider one/several of the aspects illustrated even before your own appointment? Which one(s) was it?   ⬜ No ⬜ Yes (please specify): ___________________________________________________________________________ |

| **3.** | **Specific questions about the illustrations** | Fully agree | Agree | Neutral | Disagree | Strongly disagree |
| --- | --- | --- | --- | --- | --- | --- |
|  | 1. The illustrations are comprehensible. | ⬜ | ⬜ | ⬜ | ⬜ | ⬜ |
|  | 1. The keywords used support a better understanding. | ⬜ | ⬜ | ⬜ | ⬜ | ⬜ |
|  | 1. 8. The illustrations are better to understand than pure text. | ⬜ | ⬜ | ⬜ | ⬜ | ⬜ |
|  | 1. 9. The illustrations help to prepare for an OTC. | ⬜ | ⬜ | ⬜ | ⬜ | ⬜ |
|  | 1. The OTC can be simplified by the illustrations. | ⬜ | ⬜ | ⬜ | ⬜ | ⬜ |
|  | 1. The design is appealing. | ⬜ | ⬜ | ⬜ | ⬜ | ⬜ |
|  | 1. What can be improved for the illustrations? (free answer) _________________________________________________________________________________________________________________   _________________________________________________________________________________________________________________ | | | | | |
|  |  | | | | | |
